# A resting-state network comparison of combat-related PTSD with combat-exposed and civilian controls

**DOI:** 10.1093/scan/nsz072

**Published:** 2019-10-07

**Authors:** Thomas J Vanasse, Crystal Franklin, Felipe S Salinas, Amy E Ramage, Vince D Calhoun, Paul C Robinson, Mitchell Kok, Alan L Peterson, Jim Mintz, Brett T Litz, Stacey Young-McCaughan, Patricia A Resick, Peter T Fox, STRONG STAR Consortium

**Affiliations:** 1 Research Imaging Institute, University of Texas Health Science Center, San Antonio, TX 78229, USA; 2 Department of Radiology, University of Texas Health Science Center, San Antonio, TX 78229, USA; 3 Department of Psychiatry, University of Texas Health Science Center, San Antonio, TX 78229, USA; 4 Department of Communication Sciences and Disorders, College of Health and Human Services, University of New Hampshire, Durham, NH 03824, USA; 5 The Mind Research Network, Albuquerque, NM 87106, USA; 6 Carl R. Darnall Army Medical Center, Fort Hood, TX 76544, USA; 7 Research and Development Service, South Texas Veterans Health Care System, San Antonio, TX 78229, USA; 8 Massachusetts Veterans Epidemiological Research and Information Center, VA Boston Healthcare System, Boston, MA 02130, USA; 9 Department of Psychiatry, Boston University School of Medicine, Boston, MA 02118, USA; 10 Department of Psychological and Brain Sciences, Boston University, Boston, MA 02215, USA; 11 Department of Psychiatry and Behavioral Sciences, Duke University Medical Center, Durham, NC 27707, USA; 12 Department of Electrical and Computer Engineering, University of New Mexico, Albuquerque, NM 87131, USA; 13 Department of Psychology, University of Texas, San Antonio, TX 78249, USA; 14 Department of Epidemiology and Biostatistics, University of Texas Health Science Center, San Antonio, TX 78229, USA; 15 Tri-institutional Center for Translational Research in Neuroimaging and Data Science (TReNDS), Georgia State University 30302, Georgia Institute of Technology, Emory University 30322, Atlanta, GA, USA

**Keywords:** PTSD, resting-state, stress, combat, military

## Abstract

Resting-state functional connectivity (rsFC) is an emerging means of understanding the neurobiology of combat-related post-traumatic stress disorder (PTSD). However, most rsFC studies to date have limited focus to cognitively related intrinsic connectivity networks (ICNs), have not applied data-driven methodologies or have disregarded the effect of combat exposure. In this study, we predicted that group independent component analysis (GICA) would reveal group-wise differences in rsFC across 50 active duty service members with PTSD, 28 combat-exposed controls (CEC), and 25 civilian controls without trauma exposure (CC). Intranetwork connectivity differences were identified across 11 ICNs, yet combat-exposed groups were indistinguishable in PTSD *vs* CEC contrasts. Both PTSD and CEC demonstrated anatomically diffuse differences in the Auditory Vigilance and Sensorimotor networks compared to CC. However, intranetwork connectivity in a subset of three regions was associated with PTSD symptom severity among executive (left insula; ventral anterior cingulate) and right Fronto-Parietal (perigenual cingulate) networks. Furthermore, we found that increased temporal synchronization among visuospatial and sensorimotor networks was associated with worse avoidance symptoms in PTSD. Longitudinal neuroimaging studies in combat-exposed cohorts can further parse PTSD-related, combat stress-related or adaptive rsFC changes ensuing from combat.

## Introduction

Military service members and veterans who have experienced extreme trauma in military combat are especially vulnerable to post-traumatic stress disorder (PTSD) (Kilpatrick *et al.,* 2013). Of those who served in Operation Enduring Freedom or Operation Iraqi Freedom (OEF/OIF), the population of interest in this study, disease prevalence has been estimated at 23% (Fulton *et al.,* 2015). Psychotherapy is most effective in treating individuals with PTSD (Institute of Medicine, 2014; Kirkpatrick & Heller, 2014); however, between 20 and 50% of patients do not respond to first-line treatments (Schottenbauer *et al.,* 2008). This therapeutic gap requires an improved, neurobiological understanding of PTSD (Insel *et al.,* 2010). To date, functional neuroimaging assessments of PTSD patients have largely relied on threat-related, emotional processing task paradigms, which has led to a theorization of ‘top-down’ dysregulation in PTSD from pre-frontal connections to the amygdala with hypo- and hyperactivity, respectively, resulting in exaggerated fear response (Rauch *et al.,* 2006; Patel *et al.,* 2012). Alternatively, resting-state functional connectivity (rsFC) provides a stable and generalizable perspective (Gratton *et al.,* 2018) of the neurobiological mechanisms underlying psychopathology at baseline, or ‘rest’ (Menon, 2011).

Large-scale intrinsic connectivity networks (ICNs) encompass brain regions that coherently and spontaneously fluctuate in blood-oxygenation level-dependent (BOLD) signal as measured from resting-state functional magnetic resonance imaging (rs-fMRI) (Beckmann *et al.,* 2005). Many ICNs are spatially similar both at rest and during an explicit task (Smith *et al.,* 2009), and each serves specialized cognitive, motor, sensory and interoceptive functions when ‘activated’ (Laird *et al.,* 2011). The Default-mode network is associated with self-related thoughts often concerning the past or future (Laird *et al.,* 2009), and multiple rsFC studies have reported PTSD-related differences among the posterior cingulate and hippocampus/parahippocampus nodes (Bluhm *et al.,* 2009; Sripada *et al.,* 2012a; Ashley C Chen, 2013; Miller *et al.,* 2017). Abnormal rsFC in the Salience network, which helps direct attention to relevant internal or external stimuli, has also been demonstrated (Rabinak *et al.,* 2011; Sripada *et al.,* 2012a; Tursich *et al.,* 2015; Zhang *et al.,* 2016b). In addition, diminished network segregation (i.e. increased functional network connectivity, or FNC) between ICNs during resting state has been reported (Sripada *et al.,* 2012a; Jin *et al.,* 2013; Koch *et al.,* 2016; Miller *et al.,* 2017).

While some studies have already reported rsFC differences associated with PTSD (for review, see Koch *et al.* 2016), there are noteworthy limitations within this burgeoning research. First, many experiments have not evaluated ICNs beyond those that are cognitively related, namely the Default-mode, Salience, Executive and Fronto-Parietal networks (Smith *et al.,* 2009). Ignoring other networks overlooks a large repertoire of rsFC dynamics that contribute to brain organization and, potentially, the development and maintenance of PTSD as recently suggested (Zhang *et al.,* 2015; Misaki *et al.,* 2018). Second, most studies have applied seed-based (i.e. *a priori* region-of-interest) analyses to study disease effects within and between ICNs (Bluhm *et al.,* 2009; Daniels *et al.,* 2010; Rabinak *et al.,* 2011; Yin *et al.,* 2011; Sripada *et al.,* 2012b; Ashley C Chen, 2013; Kennis *et al.,* 2014; DiGangi *et al.,* 2016; Miller *et al.,* 2017). This methodology imposes stricter assumptions of the temporal and spatial structure of neuroimaging data compared to independent component analysis (ICA) (Calhoun & de Lacy, 2017) and thus presents problems of interpretability (Cole, 2010; Constable *et al.,* 2013). Third, many rsFC studies have not considered trauma exposure, potentially confounding effects related to PTSD and traumatic exposure. In regard to combat-related PTSD, some recent studies have suggested that military deployment (van Wingen *et al.,* 2012; Kennis *et al.,* 2015; DiGangi *et al.,* 2016; Reuveni *et al.,* 2016; Misaki *et al.,* 2018) may affect rsFC and even gray matter volume (Clausen *et al.,* 2017; Wrocklage *et al.,* 2017). The few studies that have incorporated both combat-exposed and civilian control (CC) populations have proposed that some rsFC differences could represent stress-related changes, adaptive changes from combat or pre-combat protective factors against developing PTSD (Kennis *et al.,* 2015; Misaki *et al.,* 2018).

Symptom correlations are a powerful tool to interpret rsFC differences in PTSD. Total PTSD symptoms are of interest, as well as specific symptom dimensions including arousal, reexperiencing and avoidance, which can perhaps elucidate a refined understanding of neurobiological differences observed within PTSD. Some rsFC studies have indeed investigated PTSD in this manner (Reuveni *et al.,* 2015; Tursich *et al.,* 2015; Miller *et al.,* 2017); however, they are limited in number and several have utilized relatively small sample sizes.

In this study, we performed a two-step approach. First, we applied a state-of-the-art group independent component analysis (GICA) technique—group-information-guided ICA (Du & Fan, 2013)—in resting-state fMRI of 50 treatment-seeking OEF/OIF active duty service members with PTSD, 28 controls with deployment experience and without PTSD (combat-exposed controls (CECs)) and 25 healthy CCs to examine 13 canonical resting-state networks (Smith *et al.,* 2009; Laird *et al.,* 2011). We hypothesized that intranetwork and internetwork connectivity would identify group-wise effects distinguishing combat-related PTSD, CECs and CCs. Second, we probed each region/network identified in the omnibus analysis for correlations with PTSD symptom severity to interpret our findings.

## Methods and materials

### Subject recruitment

This study was conducted at the Carl R. Darnall Army Medical Center at Fort Hood, TX, and at The University of Texas Health Science Center at San Antonio as part of the South Texas Research Organizational Network Guiding Studies on Trauma and Resilience (STRONG STAR Consortium). PTSD participants were recruited from a larger study of active duty service members seeking PTSD treatment after deployments in support of OEF or OIF (Resick *et al.,* 2015). Treatment study participants were invited to participate in the neuroimaging study, which was optional and did not affect treatment participation. Two gender- and age-matched control groups were also recruited: CECs recruited from Fort Hood and CCs without any prior military service or trauma history. This study was approved by institutional review boards at Brooke Army Medical Center, San Antonio, TX; The University of Texas Health Science Center at San Antonio; and the Veterans Affairs Boston Healthcare System, Boston, MA. Written informed consent was obtained from all participants.

Participants (PTSD/CEC/CC) were excluded from the study if they had a previous penetrating head injury or a head injury resulting in loss of consciousness (>20 min), a prior neurosurgical procedure or a history of neurological disorders. PTSD participants were diagnosed and assessed with the PTSD Symptom Scale—Interview (PSS-I) (Foa *et al.,* 1993). Furthermore, the Mini International Neuropsychiatric Interview (MINI) (Lecrubier *et al.,* 1997) was administered to assess co-morbidities in PTSD; PTSD subjects were not excluded from the study if they had a co-morbid Axis I disorder (these data are shared in Table S1). MINI was also administered to CEC and CC; any subject with an Axis I disorder in either CEC or CC was excluded from the study.

PTSD symptoms and severity were assessed using the PTSD Checklist—Stressor-Specific version (PCL-S) (Weathers *et al.,* 1996) for the PTSD group, PTSD Checklist—Military version (PCL-M) for the CEC group and PTSD Checklist—Civilian version (PCL-C) for the CC group. With each PCL version, subjects indicate the degree to which they have been bothered by 17 symptoms in the past week (1 = not at all bothered*,* 5 = extremely bothered). Combat experience was assessed by a modified version of the Deployment Risk and Resilience Inventory Combat Experiences (DRRI-C) questionnaire (Vogt *et al.,* 2008). This version is a frequency-based measure (1 = never, 5 = almost daily) of 23 stereotypical warfare experiences during deployment. The Lifetime Experience Checklist (Gray *et al.,* 2004) was administered to both combat-exposed groups (not CC), and it is a measure of exposure to potentially traumatic events that occurred throughout the subject’s lifetime.

### Image acquisition

T1-weighted and T2^*^-weighted functional (BOLD) MR data were obtained in a single scanning session. MRI data were obtained on a 3T Siemens TIM-Trio (Siemens Medical Solutions, Erlangen, Germany) using a standard 12-channel head coil as the RF receiver and the integrated circularly polarized body coil as the RF transmitter. T1-weighted images were acquired using an MPRAGE pulse sequence with TR/TE = 2200/2.83 ms, flip angle = 13°, FOV = 256 mm, slices = 208 and 0.8 mm isotropic voxel size. Functional (BOLD; T2^*^-weighted) imaging used a gradient-echo, echo-planar imaging sequence, acquiring 43 slices, with TR/TE = 3000/30 ms, flip angle = 90°, 2 × 2 × 3 mm spatial resolution, FOV = 256 mm and acquisition time = 10 min and 9 s (three dummy scans were acquired/discarded to handle T1-equilibrium effects).

### Image preprocessing

Preprocessing of the BOLD data was carried out using FSL’s FEAT (FMRI Expert Analysis Tool) Version 6.00. Each functional image was realigned to the middle time point using MCFLIRT (Jenkinson *et al.,* 2002), the brain was extracted using BET (Smith, 2002) and data were linearly transformed to Montreal Neurological Institute (MNI) standard space with a two-stage registration: first to individual structural space and then to standard MNI space using FLIRT (Jenkinson & Smith, 2001). Images were spatially smoothed with a Gaussian kernel of 6 mm full width at half maximum (FWHM), and voxels were resliced to 3 × 3 × 3 mm. Four subjects were excluded for motion or other image acquisition reasons (see Supplementary Material).

### ICA

Group ICA (GICA) (Calhoun *et al.,* 2001; Calhoun & Adali, 2012) was performed using the Group ICA fMRI Toolbox (GIFT v3.0b; http://mialab.mrn.org/software/gift/). A dimensionality of 20 ICA components (with a first PCA reduction step of *d* = 30) was chosen, as it has been shown to provide behaviorally specific and easily interpretable ICNs (Calhoun *et al.,* 2008; Smith *et al.,* 2009; Laird *et al.,* 2011; Ray *et al.,* 2013). In order to decrease the influence of random initializations involved in the ICA algorithm, we performed ICASSO (Himberg *et al.,* 2004; Sai Ma *et al.,* 2011) with 20 ICA runs followed by selection of the most reliable ICA run. Individual subject maps and time courses were reconstructed using the group-information-guided independent component analysis (GIG-ICA) back reconstruction approach (Du & Fan, 2013; Du *et al.,* 2015). GIG-ICA extracts subject-specific ICNs using the group-level non-artifactual components as spatial references based on a multi-objective function optimization algorithm (Du & Fan, 2013) that has been shown to outperform other back-reconstruction approaches such as spatio-temporal (dual) regression (Salman *et al.,* 2019). GIG-ICA has also proven to be more robust to motion than removing artifact/noise components per subject (Du *et al.,* 2015). Seven artifactual group-level components related to head motion, physiological noise or scanner influence were visually identified by the experimenter (TV) based on ring, non-gray matter and high temporal frequency features (Figure S1) (Pruim *et al.,* 2015).

### Identifying significant discriminatory regions (SDRs)

Based on the subject-specific ICNs, we investigated intranetwork connectivity differences of each ICN across the three pairs of groups (CC *vs* PTSD, CEC *vs* PTSD and CEC *vs* CC) to identify group-wise patterns of connectivity. In this study, we focused on investigating group differences in voxels associated with positive *z*-scores (within each component) for simplicity; i.e. we did not evaluate regions showing non-significant correlation or anti-correlation within an ICN. Toward this end, we first performed a voxel-wise, right-tailed, one-sample *t*-test (*P* < 0.05 with Bonferroni correction) to the *z*-scores from the subject-specific ICNs of all 103 subjects. Each significant voxel provided an ICN-specific network mask. For every non-artifactual ICN, a voxel-wise, 2-tailed, 2-sample *t*-test (*P* < 0.001) was performed on the *z*-scores within the network-specific mask for the three pairs of groups. If one significant region overlapped with another (e.g. PTSD>CC & CEC>CC), the largest region was exclusively considered the SDR. If two clusters with oppositely directed patterns among the same subject group (e.g. PTSD>CC & PTSD<CC) each overlapped with the same cluster with a single pattern (e.g. CEC>CC), then two distinct SDRs were identified. To correct for multiple comparisons across voxels, a spatial extent cluster-level threshold was applied using a Monte-Carlo noise simulation strategy for each network mask (Ledberg *et al.,* 1998); for more information regarding this step, see Supplementary Material. We report significance both at cluster-level *P* < 0.05, and with an additional Bonferroni correction for the number of components (cluster-level *P* < 0.05/13). In a supplementary analysis, we also investigated the influence of Wechsler Abbreviated Scale of Intelligence (WASI) IQ score as a nuisance covariate in identifying SDRs.

Of note, our study was strictly interested in finding uni-directional differences in connectivity between groups. If a spatially contiguous cluster contained both significantly higher and lower voxel values in one subject group compared to another, the pairwise *t*-test analysis would identify and distinguish these patterns with two SDRs. Other mass-univariate approaches (e.g. analysis of variance; ANOVA) would not take this information into account and thus were not applied.

### Internetwork connectivity

A regression model was used to estimate the corresponding time courses of the individual ICNs for each subject’s fMRI data (Du *et al.,* 2015). Subject-specific time courses were detrended and de-spiked, then filtered using a fifth-order Butterworth low-pass filter with a high-frequency cutoff of 0.15 Hz within GIFT. Pair-wise correlations (Fisher *r*-to-*z*) of the component time courses were calculated from non-artifactual components. ANOVA tests were performed for each pair-wise combination of networks with at least one SDR, and group differences were deemed significant if the *F* statistic remained significant after Bonferroni correction for multiple comparisons across network pairs at *P* = 0.05, corrected.

### Post hoc connectivity correlation

After the identification of SDRs and FNCs in an omnibus analysis, we used the mean *z*-score of voxels within each SDR and FNCs to discriminate correlative effects with PTSD symptom severity. We performed correlations combining both CEC and PTSD subjects (PCL), as well as in PTSD only for specific symptom clusters including reexperiencing, avoidance and arousal (PSS-I). Because the PCL score showed a clear bimodal distribution (excess kurtosis: −1.48) in combined CEC and PTSD cohorts, we applied a non-parametric Spearman’s rank-order correlation in that specific instance to handle bias associated with Pearson’s *r* (Bishara & Hittner, 2014). In all other correlations, Pearson’s *r* was applied. All correlations and plots were performed in R v3.3.0 (R Core Team, 2016) with the ggplot2 (Wickham, 2009) package. In a supplementary analysis, all significant correlations were further tested as partial correlations after controlling for age, gender and IQ with the ppcor package (Kim, 2015). Also, SDR correlation with BDI was investigated.

### Data availability

Statistical image data (unthresholded GICA components and *t*-stat images) are currently shared as a collection at NeuroVault (https://identifiers.org/neurovault.collection:5007) (Gorgolewski *et al.,* 2015). All neuroimaging data used for the analyses reported here are stored in raw, anonymized format on an XNAT-based server (Marcus *et al.,* 2007) at the Research Imaging Institute. Data-use requests will be entertained and should be addressed to the senior corresponding author (P.T.F.).

## Results

### Subjects

Table 1 summarizes subject demographics after exclusionary criteria were applied. While PTSD and CEC groups served an equivalent number of OEF/OIF deployments (*P* = 0.45), self-reported combat experiences as measured by DRRI-C were significantly higher (*P* = 0.003) in PTSD (53 ± 16) compared to CEC (43 ± 15); the baseline score of DRRI-C is 23, which indicates no stereotypical combat experience. Current Axis I co-morbidities for PTSD are reported in Table S1. While CC subjects were excluded if they were taking a psychotropic medication, medication status did not affect study enrollment for combat-exposed groups; 58 and 3% of PTSD and CEC subjects were taking a psychotropic medication at the time of the study, respectively.

**Table 1:** Subject demographics. Abbreviations: BAI, Beck Anxiety Inventory; BDI, Beck Depression Inventory; W/B/AI/PI/O, White, Black, American Indian, Pacific Islander, Other (includes Hispanic ethnicity); DRRI, Deployment Risk and Resilience Inventory; fMRI, functional magnetic resonance imaging; OEF, Operation Enduring Freedom; OIF, Operation Iraqi Freedom; PCL-C, PTSD Checklist—Civilian version; PCL-M, PTSD Checklist—Military version; PCL-S, PTSD Checklist—Stressor-Specific version; PTSD, posttraumatic stress disorder

	**Posttraumatic stress disorder (PTSD)**	**Combat-exposed controls (CEC)**	**Civilian controls (CC)**	**Group Comparison**
*N*	50	28	25	-
Age	33 ± 8.2	32 ± 5.8	32 ± 10.6	F_(2,100)_ = 0.25, *P* = 0.77
Race				-
White	66%	64%	76%	
Black	22%	21%	16%	
American Indian	2%	0%	0%	
Pacific Islander	2%	0%	0%	
Other	8%	14%	8%	
Gender				χ^2^ = 0.45, *P* = 0.80
Male	92%	93%	88%	
Female	8%	7%	12%	
PTSD Checklist (PCL-S,M,C) score	56 ± 12.9	19 ± 3.2	19 ± 2.8	F_(2,100)_ = 200, *P* < 0.001
BDI score	28.2 ± 12.2	2.1 ± 3.3	1.7 ± 2.5	F_(2,100)_ = 116, *P* < 0.001
BAI score^a^	19.6 ± 13.0	1.6 ± 2.2	1.7 ± 2.0	F_(2,99)_ = 50, *P* < 0.001
WASI IQ	97.8 ± 10.9	98.2 ± 10.9	111.2 ± 12.3	F_(2,100)_ = 3.1, *P* < 0.001
PTSD symptom score—interview (PSS-I; total)	26.6 ± 7.4	-	-	-
OEF/OIF deployments	2.4 ± 1.3	2.2 ± 1.2	-	*t*_76_ = 0.75, *P* = 0.45
Time since last deployment (months)	17.8 ± 14	23.0 ± 27	-	*U* = 654, *P* = 0.63
DRRI—combat experiences total	53.4 ± 16.2	42.6 ± 14.5	-	*t*_76_ = 2.92, *P* = 0.004
Life experience checklist^b^	5.8 ± 2.5	4.0 ± 1.7	-	*t*_75_ = 3.41, *P* = 0.001
fMRI motion (mean relative/frame-wise displacement, mm)	0.081 ± 0.035	0.071 ± 0.025	0.069 ± 0.024	*F*_(2,100)_ = 1.6, *P* = 0.20

^a^One PTSD subject did not complete the BAI inventory

^b^One PTSD subject did not complete the LEC inventory

### Components

**Fig. 1:**
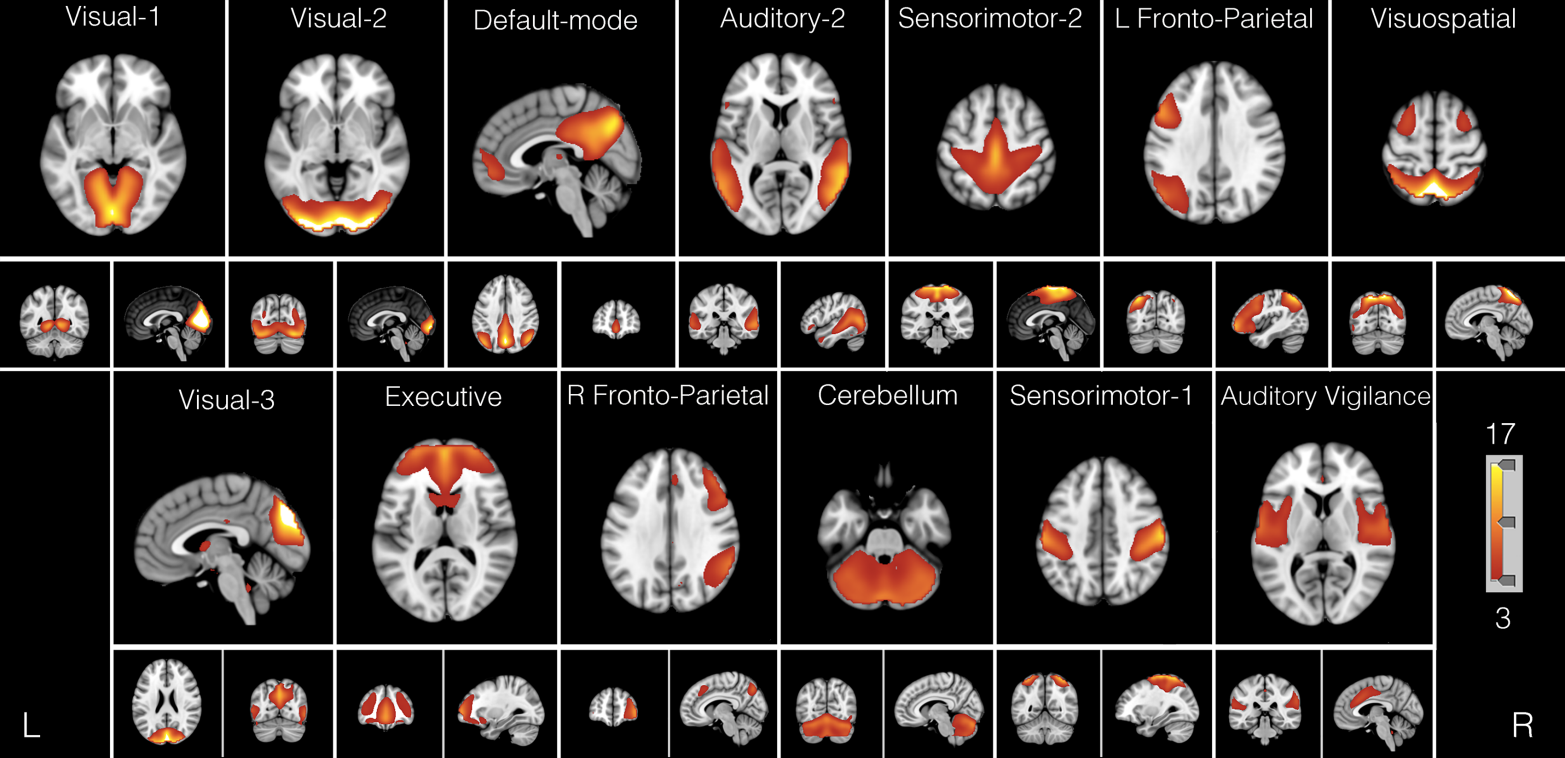
Group ICA components. Thirteen group-level, non-artifactual intrinsic connectivity networks (ICNs). Group independent component analysis (GICA) (*d* = 20) was applied to all 103 subjects. Each group ICN is thresholded at *z* > 3 and is shown with its network name. ICNs are ordered left to right and top to bottom in order of explained variance.

Thirteen non-artifactual group networks of interest are displayed in Figure 1. To identify appropriate titles of every ICN, each of the 13 networks was spatially correlated with the 10 resting-state networks reported by Smith *et al.* (2009) (Figure S2). One network (IC-8) was labeled as ‘Visuospatial’ (Laird *et al.,* 2011), as it did not correspond to any featured by Smith *et al.* (2009). The Auditory Vigilance network label is discussed in depth in the Discussion section.

### SDRs

**Fig. 2:**
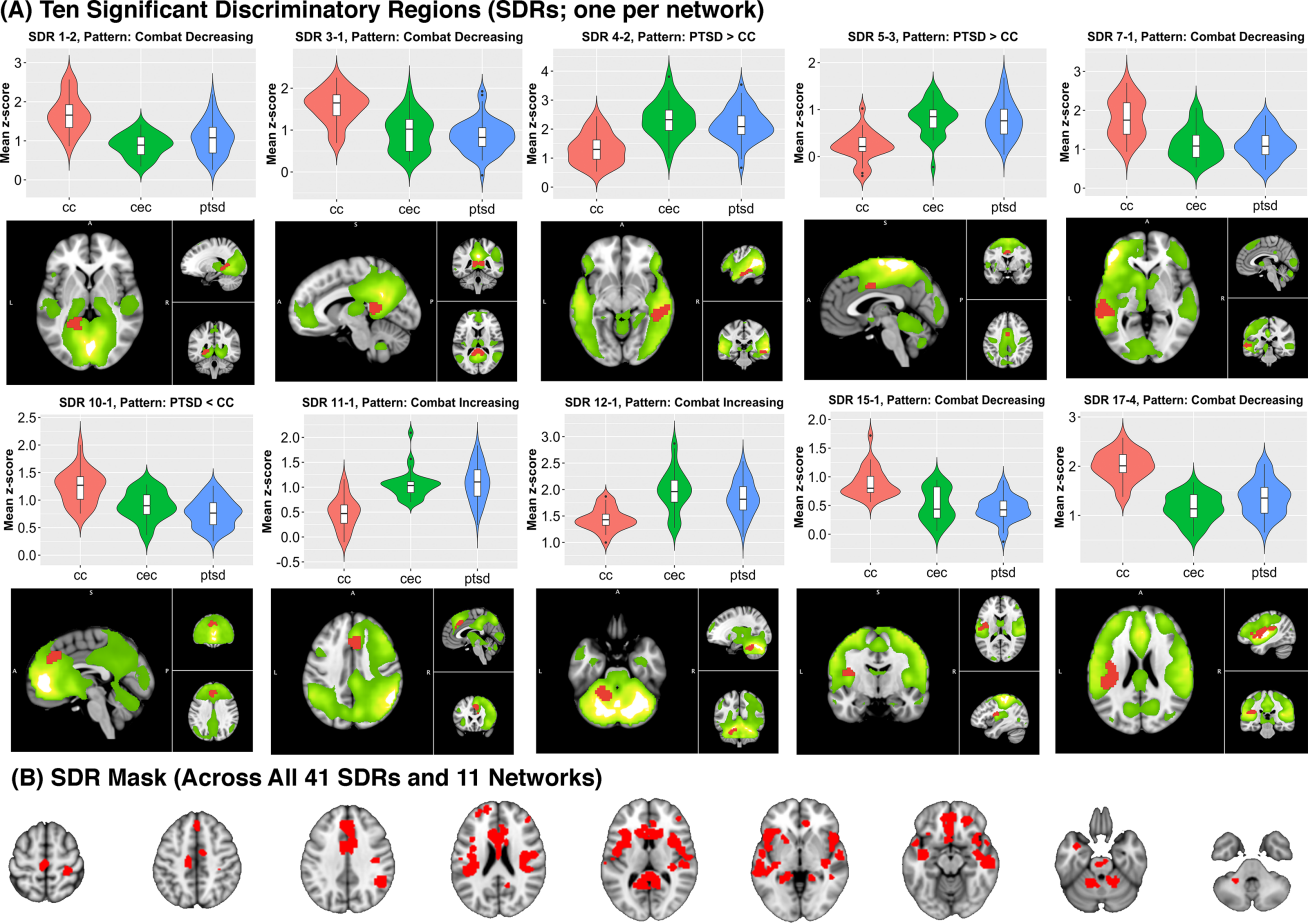
SDR patterns. (A) Ten (of 41) selected significant discriminatory regions (SDRs; red) extracted from voxel-wise, 2-sample *t*-tests of intrinsic connectivity network (ICN) spatial maps across groups (PTSD *vs* CC, PTSD *vs* CEC and CEC *vs* CC). SDR M-N (e.g. SDR 1–2) corresponds to the Mth ICN (ordered by explained variance) and the Nth SDR (arbitrarily ordered) within the Mth ICN. Violin & boxplots show the mean SDR *z*-score distribution per group, which represents connectivity within that ICN. The green overlay is the positive *t*-stat images for each ICN, masked at Bonferroni-corrected *P* = 0.05. CC indicates civilian controls; CEC, combat-exposed controls; PTSD, posttraumatic stress disorder. (B) Conjunction image of all 41 SDR masks.

Forty-one SDRs were identified across 11 ICNs (Table 2) (cluster-forming *P* = 0.001, cluster-level *P*, uncorrected = 0.05). Eighteen of 41 SDRs (44%) survived a more stringent significance threshold (Table 2, Column 11) applying Bonferroni correction for the number of non-artifactual components (cluster-level *P* < 0.05/13). Ten SDRs are featured (one per network) in Figure 2A. SDR patterns (e.g. ‘PTSD > CC’ or ‘Combat Increasing’) are reported in Table 2, along with peak statistical coordinates in MNI space. Only one SDR did not remain significant after including WASI IQ as a nuisance covariate and was thus not reported among significant results. If one SDR contained an overlapping PTSD > CC & CEC > CC effect, it was labeled with a ‘Combat Increasing’ pattern in Table 2. Likewise, Combat Decreasing patterns were labeled this way.

**Table 2:** Significant discriminatory regions (SDRs). Abbreviations: CC, civilian controls; CEC, combat-exposed controls; ICN, intrinsic connectivity network; PTSD, posttraumatic stress disorder; PCL, cluster-level *P* value. Max *t*-statistic coordinates are reported in standardized MNI space

**Significant discriminatory region**	**SDR pattern**	**Size (mm^3^)**	***T*-stat**	***x***	***y***	***z***	**Talairach Daemon label**			**SDR survives multiple comp. correction across 13 networks, *P*CL < 0.05/13**
**Component 1 (Visual 1)**										
1-1	Combat decreasing	1863	6.30	15	−42	3	R	Parahippocampus	BA 30	Yes
1-2	Combat decreasing	2511	5.82	−9	−54	9	L	Pos cingulate	BA 30	Yes
**Component 2 (Visual 2)**										
n/a										
**Component 3 (Default mode)**										
3-1	Combat decreasing	3375	7.41	−6	−42	12	L	Pos cingulate	BA 29	Yes
3-2	PTSD > CC	1485	−4.64	−12	42	−12	L	Med frontal gyrus	BA 10	Yes
**Component 4 (Auditory 2)**										
4-1	Combat decreasing	2295	5.24	51	−48	36	R	Supramarginal gyr	BA 40	Yes
4-2	PTSD > CC	2727	−4.40	51	−33	−6	R	Mid temporal gyr	BA 21	Yes
**Component 5 (Sensorimotor 2)**										
5-1	Combat decreasing	702	5.45	−15	−21	45	L	Cingulate gyrus	BA 31	No
5-2	PTSD < CC	594	5.05	6	−6	51	R	Med frontal gyrus	BA 6	No
5-3	PTSD > CC	999	−6.86	3	0	36	R	Cingulate gyrus	BA 24	No
5-4	CEC < CC	864	5.24	−3	−24	60	L	Paracentral lobule	BA 6	No
**Component 7 (L. fronto-parietal)**										
7-1	Combat decreasing	2295	5.59	−60	−33	3	L	Mid temporal gyr	BA 22	Yes
7-2	Combat decreasing	1269	5.28	−33	24	3	L	Insula	BA 13	No
7-3	CEC > CC	918	−4.93	−42	3	15	L	Insula	BA 13	No
**Component 8 (Visuospatial)**										
8-1	PTSD < CC	756	5.21	6	−42	9	R	Pos cingulate	BA 29	No
8-2	PTSD > CC	1161	−4.96	−42	0	−21	L	Sup temporal gyr	BA 38	No
**Component 9 (Visual 3)**										
n/a										
**Component 10 (Executive)**										
10-1	PTSD < CC	2322	5.27	−6	30	39	L	Cingulate gyrus	BA 32	Yes
10-2	PTSD < CC	1242	5.49	−39	18	−6	L	Insula	BA 13	No
10-3	PTSD > CC	1998	−6.67	0	24	−18	L	Anterior cingulate	BA 32	Yes
10-4	PTSD > CC	1161	−6.28	3	24	12	R	Anterior cingulate	BA 33	No
10-5	Combat decreasing	3024	7.37	−12	21	12	L	Caudate	Caudate	Yes
10-6	CEC > CC	1242	−4.77	−15	54	24	L	Sup frontal gyrus	BA 9	No
**Component 11 (R. fronto-parietal)**										
11-1	Combat increasing	1971	−5.89	3	18	39	R	Cingulate gyrus	BA 32	Yes
11-2	PTSD > CC	891	−6.06	6	36	6	R	Anterior cingulate	BA 24	No
11-3	Combat decreasing	1053	6.59	0	39	42	L	Sup frontal gyrus	BA 8	No
11-4	CEC < CC	837	5.74	24	42	−12	R	Mid frontal gyrus	BA 11	No
**Component 12 (Cerebellum)**										
12-1	Combat increasing	1863	−6.40	−24	−45	−33	L			Yes
12-2	Combat increasing	1431	−5.02	15	−48	−24	R	Culmen		No
12-3	CEC > CC	756	−4.46	−3	−24	−24	L			No
**Component 15 (Sensorimotor 1)**										
15-1	Combat decreasing	1485	6.08	−39	−6	18	L	Insula	BA 13	Yes
15-2	PTSD < CC	567	4.65	45	42	15	R	Mid frontal gyrus	BA 46	No
15-3	CEC < CC	972	5.70	30	−33	60	R	Sub-gyral	BA 40	No
15-4	CEC < CC	567	5.84	36	−9	18	R	Insula	BA 13	No
15-5	CEC > CC	783	−5.63	51	−21	6	R	Sup temporal gyr	BA 13	No
**Component 17 (Auditory vigilance)**										
17-1	PTSD < CC	1053	5.55	33	12	−18	R	Inf frontal gyrus	BA 13	No
17-2	Combat increasing	2727	−7.21	33	−27	6	R	Insula	BA 13	Yes
17-3	Combat increasing	1242	−5.93	42	−15	30	R	Precentral gyrus	BA 6	No
17-4	Combat decreasing	9207	7.40	−36	−12	21	L	Insula	BA 13	Yes
17-5	Combat decreasing	8721	7.61	39	−6	18	R	Insula	BA 13	Yes
17-6	Combat increasing	7641	−6.48	−9	15	24	L	Cingulate gyrus	BA 24	Yes
17-7	Combat increasing	5373	−7.06	−39	−12	−15	L	Sub-gyral	BA 21	Yes
17-8	CEC > CC	1296	−5.14	57	−9	0	R	Sup temporal gyr	BA 22	Yes

### Internetwork connectivity

**Fig. 3:**
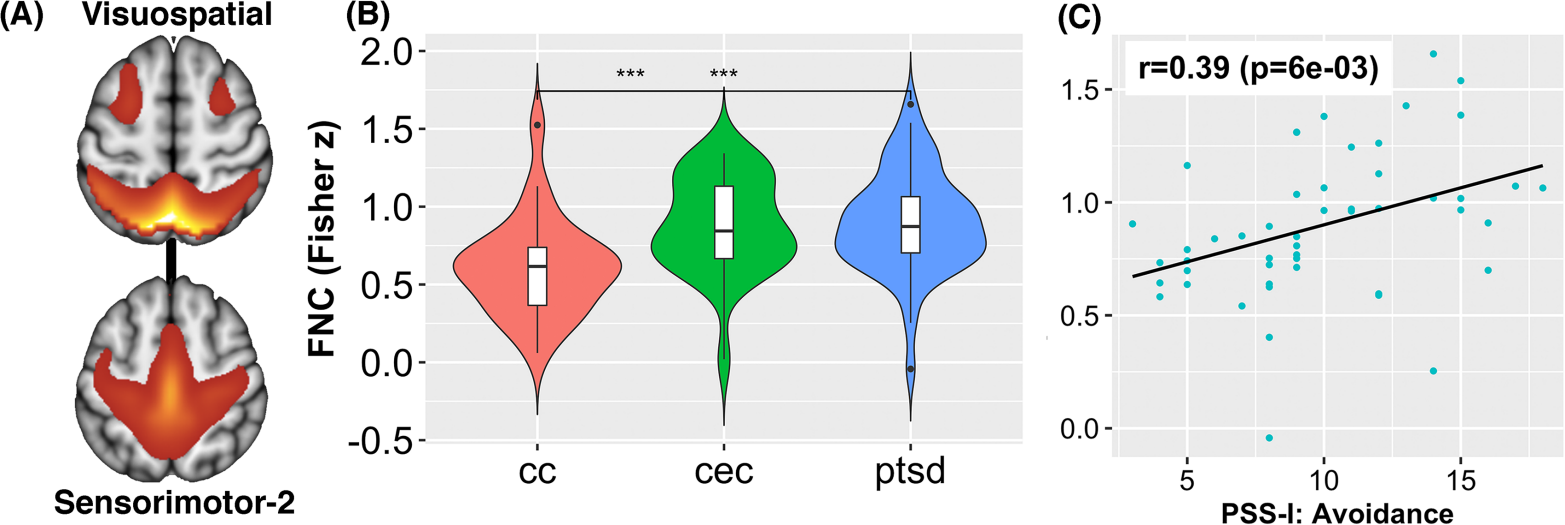
Functional network connectivity (FNC). A significant FNC network pair that included (A) the Sensorimotor-2/Visuospatial networks: (B) *F*_2,100_ = 7.57 (*P* = 0.049, corrected). Post hoc *t* tests found significant group differences in PTSD *vs* CC and CEC *vs* CC, but not PTSD *vs* CEC. (C) FNC within the PTSD group showed significant correlation with avoidance symptoms.

One FNC group difference was significant at *P* < 0.05 (corrected) and is displayed in Figure 3. This network pair included the Sensorimotor-2 and Visuospatial networks (*F*_2,100_ = 7.57; *P* = 0.049, corrected). Post hoc *t*-tests demonstrated significant group differences in PTSD *vs* CC (*P* = 0.001, corrected), PTSD *vs* CEC (*P* = 0.006, corrected), but not PTSD *vs* CEC (*P* = 0.84, corrected).

### Connectivity correlations

**Fig. 4:**
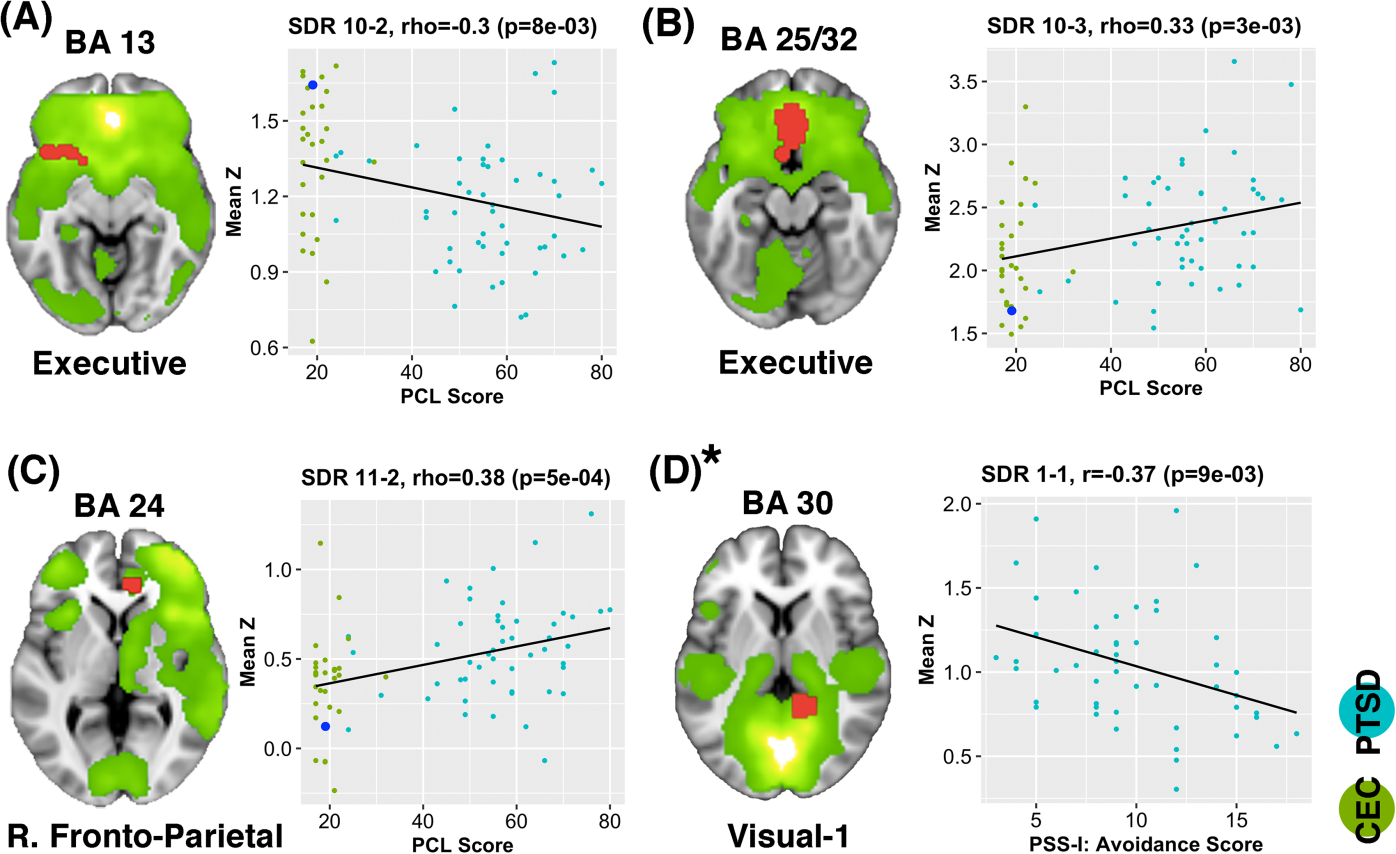
Connectivity correlations with inventories. (A–C) Spearman rho correlations (*P* < 0.01) of mean *z*-scores within a significant discriminatory region (SDR) with PTSD checklist (PCL) scores among combined combat-exposed controls (CECs) and posttraumatic stress disorder (PTSD) cohorts. Green dots correspond to CEC; teal dots correspond to PTSD; large blue dots correspond to the mean *z*-score of CC. (D) Pearson correlation (*P* < 0.01) of the PTSD Symptom Scale—Interview (PSS-I) symptom cluster score (avoidance, arousal, or reexperiencing) and mean *z*-score within the PTSD cohort only. BA indicates Brodmann area. *SDR 1–1 correlation exceeded the tested significance threshold (*P* > 0.01) after adjustment for age, gender and IQ (*P* = 0.029).

Four SDRs correlated with PTSD symptoms at the tested significant threshold (*P* = 0.01) (Figure 4). However, SDR 1–1 exceeded this significance threshold after adjusting for age, gender and IQ (*P* = 0.029). FNC within the PTSD group showed significant correlation (*r* = 0.39; *P* = 0.006) with avoidance symptoms in the Sensorimotor-2/Visuospatial networks (Figure 3C). This effect did not change after adjusting for age, gender or IQ. In addition to PCL scores, after controlling for age, gender and IQ, SDRs 10–2 (*P* = 0.009) and SDR 11–2 (*P* = .010) also correlated with BDI scores. Outside of these regions, only SDR 12–3 correlated with BDI scores after adjusting for age, gender and IQ (rho = −0.34; *P* = 0.002).

## Discussion

**Fig. 5:**
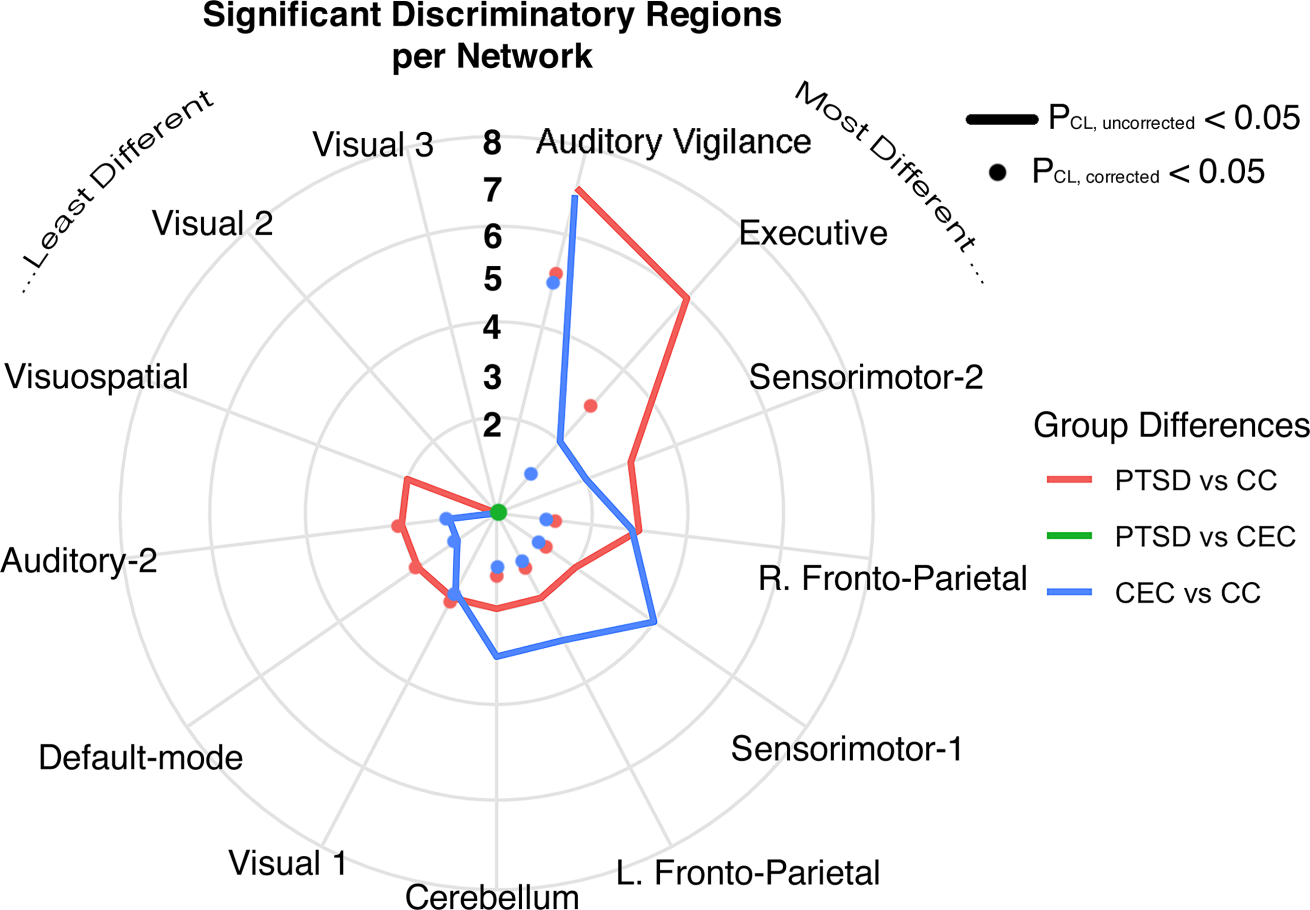
Intranetwork connectivity patterns. Spider plot displaying the number of significant discriminatory regions (SDRs) identified for each intrinsic connectivity network (ICN) derived from 2-sample *t*-tests of ICN spatial maps across three pairs of groups (posttraumatic stress disorder (PTSD) *vs* combat-exposed controls (CECs), posttraumatic stress disorder *vs* civilian controls (CC) and combat-exposed controls *vs* civilian controls); 41 SDRs were identified in total. Lines show all SDRs; dots show only SDRs that survive correction for multiple comparisons across 13 components. Group differences according to the SDR pattern within each SDR are color-coded. Numerical labels 2–8 indicate the number of SDRs identified per ICN. Of note, no significant (corrected or uncorrected) PTSD *vs* CEC patterns were identified (green dot). PCL—cluster-level *P* value.

Our prediction of group differences in rsFC across military service members with PTSD, service members without PTSD and CCs in ICNs was confirmed; a total of 41 SDRs were identified across 11 (of 13) ICNs. Contrary to our hypothesis, group patterns in SDRs were entirely amongst service members [PTSD *vs* CC, CEC *vs* CC or (PTSD & CEC) *vs* non-service members (CC)]; no SDR patterns included significant differences between PTSD and CEC at the tested, voxel-wise threshold (Table 2, Figure 5). However, connectivity of three SDRs correlated with PTSD symptom scores even after adjustment for age, gender and IQ. Notably, these SDRs were within ICNs centrally important for higher cognitive function and general psychopathology: Executive and Fronto-Parietal networks (Menon, 2011). Finally, we report a pair of ICNs, the Visuospatial and Sensorimotor-2 networks, that were hyperconnected in both PTSD and CEC compared to CC. Their connectivity was associated with a specific PTSD symptom cluster in the PTSD cohort. Taken together, these findings contribute to growing neurobiological evidence expressing the importance of including CECs when studying rsFC in combat-related PTSD. Similar rsFC differences in veterans with and without PTSD compared to non-trauma-exposed controls have only recently been reported (Kennis *et al.,* 2015; DiGangi *et al.,* 2016; Reuveni *et al.,* 2016; Misaki *et al.,* 2018). It is plausible that many of our observed effects are due to combat-related stress, and some may even be reversible with sufficient time after combat (van Wingen *et al.,* 2012).

### Intranetwork connectivity

The Auditory Vigilance network contained the most intranetwork connectivity group differences of any ICN (Figure 5); effects were largely shared among both combat-exposed groups compared to CC (i.e. Combat Increasing or Combat Decreasing SDR patterns; Table 2). In addition to the primary auditory cortex, this network also included other brain areas (dorsal anterior cingulate, insula) which are typically considered to be core aspects of the ventral attention network (Yeo *et al.,* 2011) or salience network (Menon, 2015). Our network label, Auditory Vigilance, was chosen due to this broader group network (i.e. auditory cortices and ventral attention network) being activated during auditory oddball tasks (see meta-analysis of 49 fMRI experiments by Kim *et al*. (2014)). Atypical connectivity in both the Auditory Vigilance/Auditory-2 ICNs among service members may indeed reflect auditory deficits. Noise and head trauma increase the likelihood during or after deployment of developing tinnitus (Yurgil *et al.,* 2016), the most common service-connected disability as of 2016, followed by hearing loss (Department of Veterans Affairs, 2016). This study did not measure tinnitus symptoms or auditory function in participants. Multiple experiments have reported distributed rsFC abnormalities in patients with tinnitus (Maudoux *et al.,* 2012; Feng *et al.,* 2018); one such study showed increased rsFC between the auditory cortex and dorsal anterior cingulate correlated with disease duration (Chen *et al.,* 2018). Both regions were key nodes and hyperconnected within the greater Auditory Vigilance network in this analysis (SDRs: 17-6 and 17-8).

In the Executive network, 6 SDRs were identified. Four of these SDRs had a specific PTSD *vs* CC pattern (Table 2). This network includes several medial–frontal areas, the caudate and the ventral anterior insula. It supports multiple cognitive functions, as well as action–inhibition, emotion and self-referential processing (Smith *et al.,* 2009). One SDR was present in the ventral anterior cingulate (SDR:10-3), and stronger network coupling correlated with PTSD symptom severity. Multiple studies have shown resting-state hyperactivity (Koch *et al.,* 2016) among PTSD subjects in this brain area, and hyperconnectivity in this region has been previously associated with a maladaptive stress response (Thomason *et al.,* 2011). The left ventral anterior insula (SDR:10-2) finding—which does not survive multiple-comparison correction across components—does support one prior non-combat-related PTSD study (Zhang *et al.,* 2016a). The dorsal anterior cingulate also showed decreased Executive network connectivity (SDR:10-1). This finding could be further evidence of a common neurobiological substrate (anterior insula and dorsal anterior cingulate), or ‘endophenotype’, that may underlie similar cognitive symptoms observed across psychiatric disorders as proposed in multiple structural and rsFC meta-analyses (Goodkind *et al.,* 2015; Sha *et al.,* 2018; Vanasse *et al.,* 2018). Supporting such an interpretation, SDR 10-2 correlated with BDI scores (*P* = 0.009) in addition to PCL scores. The co-morbidity rate of major depressive disorder (MDD) is especially high among PTSD patients (48–55%) (Elhai *et al.,* 2008), a fact consistent with the sample investigated here (56%).

Left- and right-lateralized Fronto-Parietal ICNs are associated with reasoning, attention, action inhibition and working memory (Laird *et al.,* 2011). While most cognitive assessments in PTSD have been cross-sectional (Aupperle *et al.,* 2012), a wealth of literature has demonstrated decreased performance on measures of auditory attention and working memory in combat- and sexual assault-related PTSD compared to controls (Brandes *et al.,* 2002; Samuelson *et al.,* 2006; LaGarde *et al.,* 2010). A previous ICA assessment found increased intranetwork connectivity of the dorsal anterior cingulate within the Fronto-Parietal network among PTSD patients during a subliminal threat-related task (Rabellino *et al.,* 2015). The authors suggested that a Fronto-Parietal network re-organization (which overengages medial frontal areas) could perhaps account for worse cognitive control. Our analysis implicated two hyperconnected Fronto-Parietal regions within the anterior cingulate (SDRs:11-1,11-2). Also, SDR:11-2 showed a strong correlation to symptom scores using the PCL (Spearman’s rho = 0.38) across CEC and PTSD groups (Figure 4c).

The Default-mode network contained two SDRs, both of which survived more stringent cluster-level thresholds. The medial anterior aspect of the Default-mode network (SDR:3-2) was hyper-connected in PTSD, with a PTSD>CC connectivity pattern. The posterior cingulate (SDR:3-1) showed a Combat Decreasing connectivity pattern. The Default-mode network has arguably garnered the most attention in PTSD rsFC research to date because of its involvement in self-referential processing and its focus in early work (Bluhm *et al.,* 2009), but our data-driven analysis did not reveal it to be at the forefront of ICN differences (Figure 5).

SDR 1-1, which included the right parahippocampus and extended into the posterior cingulate, was negatively correlated with avoidance symptoms (Pearson’s *r* = −0.37) in PTSD, however, this association exceeded the tested significance threshold after adjusting for age, gender and IQ (*P* = 0.029). As previously mentioned, disrupted rsFC of the parahippocampus and posterior cingulate have been widely reported, most often in the context of Default-mode network connectivity (Bluhm *et al.,* 2009; Sripada *et al.,* 2012a; Ashley C Chen, 2013; Miller *et al.,* 2017; Misaki *et al.,* 2018). SDR 1-1 lies within the retrosplenial cortex, an area that supports specific location identification (as opposed to categorical scene identification like, ‘store’ or ‘park’), and allows scenes to be localized within an extended environment (Epstein & Higgins, 2007). While SDR 1-1’s correlation was marginally affected after adjusting for confounding variables, Miller *et al.* (2017) also found that right parahippocampus rsFC was negatively associated with avoidance symptoms. SDR 1-1 could represent a mechanism by which contextual fear extinction processes are impaired in PTSD.

Adaptive rsFC changes in response to combat, or pre-combat protective factors that promote resiliency in soldiers may underlie some regions reported here. Some task activation and rsFC studies have demonstrated differences in veterans without PTSD compared to CCs (New *et al.,* 2009; Blair *et al.,* 2012; Kennis *et al.,* 2015; Misaki *et al.,* 2018). Some areas reported by Misaki *et al.* (2018) in a similar three-group rsFC analysis include the right transverse temporal gyrus (SDR:15-5) and left superior frontal (SDR:10-6) gyrus, which showed identical group differences in the present work (CEC *vs* CC). Only longitudinal analyses (i.e. pre- *vs* post-deployment) can confirm an adaptive or protective interpretation.

### Internetwork connectivity

The Sensorimotor-2 network was significantly hyperconnected to the Visuospatial network in PTSD and CEC compared to CC. Furthermore, Sensorimotor-2 & Visuospatial connectivity was significantly correlated with avoidance severity in the PTSD cohort. The Sensorimotor-2 ICN contained the supplementary motor area (BA 6), a region engaged in planning action sequences (Nachev *et al.,* 2008). Separately, the Visuospatial network is involved in the spatial orienting of attention (Vossel *et al.,* 2014). Increased connectivity between nodes of the SMA and Visuospatial areas has previously been shown to predict short-term task-automatization and efficiency increases (Mohr *et al.,* 2016). Among PTSD patients, using avoidance as a coping strategy is associated with psychological inflexibility, which is defined as ‘reduced likelihood of engaging in values-based actions due to rigid rule following and attempts to control difficult internal experiences, such as thoughts, emotions, and physical sensations’ (Bond *et al.,* 2011). Our FNC result could perhaps be explained as a neural mechanism of psychological inflexibility observed in PTSD (Miron *et al.,* 2015), where a general propensity for task-automatization may characterize rigid rule following in coping with traumatic memories.

Previous studies have also investigated internetwork connectivity in PTSD. In a seed-based rsFC analysis, Sripada *et al.* (2012b) demonstrated increased FNC between the DMN and Executive networks. Daniels *et al.* (2010) observed that individuals with PTSD may have difficulty disengaging the Default-mode and engaging Executive and Fronto-parietal networks during attention-related tasks. The present analysis did not yield any altered connection between the DMN and Fronto-parietal networks, even before correcting for multiple comparisons across ICN combinations.

### Limitations

Some study parameters (e.g. the number of components used in GICA) are adjustable, and the specific settings we chose may have influenced the observed results. Thus, the generalizability of our findings could be diminished in this regard. In addition, our analyses used DSM-IV diagnostic criterion (American Psychiatric Association and Staff, 2010), not DSM-5. DSM-5 (American Psychiatric Association, 2013) separates avoidance from emotional numbing symptoms for four symptom clusters instead of three and added depersonalization and de-realization as distinct PTSD sub-types (Black & Andreasen, 2014). Therefore, PSS-I symptom cluster correlations investigated in this paper did not detect depersonalization and/or derealization rsFC associations, and perhaps missed emotional numbing associations.

SDR correlative analyses presented here (*P* < 0.01) were not corrected for multiple comparisons across all 41 identified SDRs. This stream of results was aimed to provide the reader an interpretive explanation for each of the main, omnibus findings (i.e. SDRs). Future work can look to replicate the associative effects shared here. Finally, the Bonferroni procedure to correct for multiple comparisons across networks is not optimal in the present scenario (Table 2 Column 11) because of the dependency of voxels between components, which violates Bonferroni assumptions. Therefore, these results could be overly conservative.

## Conclusions

Recent, seed-based rsFC analyses have reported analogous group effects in both combat-related PTSD and combat-exposed veterans compared to CCs. This data-driven, omnibus analysis generally reflected those findings, especially in the Auditory Vigilance and non-cognitive networks. Many of the reported effects here are perhaps due to combat-related stress; future neuroimaging work can validate this interpretation by studying rsFC longitudinally, before and after deployment. Still, connectivity of SDRs within the Executive and Fronto-Parietal networks correlated with PTSD symptoms. The biomarker implications of this work can be realized in future studies with emergent machine learning methods. Specifically, a support vector machine could utilize discriminatory voxels within the spatial maps of GIG-ICA (i.e. SDRs) for its feature space (Salman *et al.,* 2019). Finally, one prospect stemming from this work is evaluating traditional psychotherapy or neuromodulation in normalizing the observed rsFC effects.

## Supplementary Material

scan-19-083-File009_nsz072
