# Supplementary material for: A resting-state network comparison of combat-related PTSD with combat-exposed and civilian controls
Source: Soc Cogn Affect Neurosci. 2019 Oct 7;14(9):933–45. doi: 10.1093/scan/nsz072 (PMC6917024; doi:10.1093/scan/nsz072)
Supplement: scan-19-083-File009_nsz072 [file scan-19-083-file009_nsz072.docx]

**SUPPLEMENTARY MATERIAL**

**for**

**“A Resting-State Network Comparison of Combat-Related PTSD with Combat-Exposed and**

**Civilian Controls”**

**by**

**Vanasse et al.**

**Significant Discriminatory Region**

To correct for multiple comparisons across voxels, AFNI’s (https://afni.nimh.nih.gov/) 3dFHWMx tool was used to extract the noise smoothness via spatial autocorrelation of the preprocessed fMRI data (with an additional de-trending option). This tool was recently updated to estimate noise smoothness more accurately based on a mixed-model rather than a purely Gaussian fitting function (Cox et al., 2017). Next, AFNI’s 3dClustSim tool was applied (with the median noise smoothness estimation parameters from the previous step with 10,000 iterations) to create a noise distribution which provided a minimum cluster-level threshold of p=0.05—with a cluster-forming p=0.001 recommended by Cox et al. (2017)—for each masked ICN network.

**Subject Exclusion Criteria (Image acquisition)**

One subject (PTSD) did not complete an anatomical scan and was excluded from further analysis. One subject (PTSD) was excluded because of global MRI signal dropout. Three subjects (2 CC and 1 PTSD) demonstrated excessive motion based on previous recommendations (Parkes et al., 2018); their mean relative displacement parameters were found to be beyond acceptable limits for this study (>0.21 mm) and they were excluded .

**

**

**Sup. Figure 1.** **Artifactual Components.** Anatomical layout of each group component identified as artifactual.

**Sup. Figure 2.** Spatial correlation matrix of the group components extracted here (d = 20) with the 10 featured by Smith et al. (2009), which was used to assign network labels.

**Sup. Table 1.** Co-morbidities among PTSD Subjects.

**References:**

Cox, R.W., Chen, G., Glen, D.R., Reynolds, R.C., Taylor, P.A., 2017. FMRI Clustering in AFNI: False-Positive Rates Redux. Brain Connect 7, 152–171. doi:10.1089/brain.2016.0475

Du, Y., Fryer, S.L., Lin, D., Sui, J., Yu, Q., Chen, J., Stuart, B., Loewy, R.L., Calhoun, V.D., Mathalon, D.H., 2018. Identifying functional network changing patterns in individuals at clinical high-risk for psychosis and patients with early illness schizophrenia: A group ICA study. Neuroimage Clin 17, 335–346. doi:10.1016/j.nicl.2017.10.018

Fisher, R.A., 1925. Statistical Methods for Research Workers. Oliver and Boyd, London.

Parkes, L., Fulcher, B., Yücel, M., Fornito, A., 2018. An evaluation of the efficacy, reliability, and sensitivity of motion correction strategies for resting-state functional MRI. Neuroimage 171, 415–436. doi:10.1016/j.neuroimage.2017.12.073

Winkler, A.M., Webster, M.A., Brooks, J.C., Tracey, I., Smith, S.M., Nichols, T.E., 2016. Non-parametric combination and related permutation tests for neuroimaging. Hum Brain Mapp 37, 1486–1511. doi:10.1002/hbm.23115
